# Monolayers of Linear Saturated Succinate Polyesters at Air-Liquid Interfaces^1^,^2^

**DOI:** 10.6028/jres.065A.003

**Published:** 1961-02-01

**Authors:** Wendell M. Lee, J. Leon Shereshefsky, Robert R. Stromberg

## Abstract

The surface pressure-area isotherms at 25 °C are reported for some linear saturated polyesters spread as monolayers at air-aqueous interfaces. Monolayers of poly (ethylene succinate), poly (pentamethylene succinate), and poly (neopentyl succinate) were studied on distilled water and 0.01 *N* hydrochloric acid subphases.

Poly (ethylene succinate) monolayers are in the expanded state and collapse at low surface pressures. Poly (pentamethylene succinate) monolayers are also expanded, but show no definite collapse, even at the highest surface pressures studied. Monolayers of poly (neopentyl succinate) collapse at high surface pressures, and are less expanded, over the entire surface pressure range studied, than the monolayers of the other polymers studied. The specific area for each polymer and the effect of structure on the surface pressure-area isotherms of poly (pentamethylene succinate) and poly (neopentyl succinate) are discussed.

## 1. Introduction

In recent years the monolayer properties of synthetic polypeptides and synthetic polymers have received considerable attention. The interest in these synthetic macromolecules derives from numerous structural variations that may be realized by proper choice of reactants and polymerization conditions. Numerous surface film studies have been made using both addition- and condensation-type polymers, such as the polyalkylacrylates, polyamides, and polyvinyl compounds. Little work, however, has been reported on the high surface-pressure properties of linear saturated polyester monolayers. Studies by Harkins, Carmen, and Ries [1]^4^ using the self ester of *ω*-hydroxydecanoic acid and by Moss [2] on poly (ethylenesuccinate) have been published.

This paper considers the surface film properties of three linear saturated polyesters of succinic acid spread as monolayers at the air-liquid interface. The spreading characteristics of these films are discussed to show the effect of solvent and solution concentration on the spreading characteristics of one of the polyester films.

## 2. Experimental Procedure

The polyesters, poly (ethylene succinate), poly-(pentamcthylene succinate), and poly (neopentyl succinate), were supplied by flames Farr, Jr. of the Research Laboratory of the Thiokol Chemical Co. They were synthesized from the melt without catalyst, using succinic acid and the appropriate glycols, and were crystalline at room temperature (X-ray diffraction). Their number-average molecular weights, as determined by end group analysis were of the order of 4500, with average degrees of polymerization of 24 to 31. These polymers were further purified by reprecipitation from chloroform solution with ethyl ether and dried in vacuum for 50 hr at 50 °C. The materials were then stored under moisture-free conditions and exposed to the atmosphere only while removing samples. Some of the properties of these polymers are given in table 1.

A Langmuir-Adam horizontal balance was used to study the film properties. The horizontal balance, very similar to that described in detail by Langmuir [3] and Adam [4], consisted of a bronze tray 65 cm long and 14 cm wide provided with leveling screws, a linear scale on one side, and a movable glass barrier $\frac{1}{2}\times \frac{1}{4}\times 12$ in. resting on the edges of the tray. The tray and barrier were coated with paraffin, the tray filled with water, and the polymer spread on the water surface between the barrier near one end of the tray and a detachable torsion balance device near the other end. This device consisted of a mica float resting on the surface of the water attached to the edges by thin polytetrafluoroethylene strips which had been sliced with a clinical microtome. The float was attached to a wire, which was attached at one end to a pointer. The movement of the pointer was magnified optically and after calibration indicated the torque caused by a force applied to the mica float. Compression of the film on the water or other subphase surface between the movable barrier and the mica float permitted measurement of differences between the surface tension of the subphase and that of the film, for a known quantity of polymer and area of film.

The balance was enclosed in a case and the assembly housed in a room held at 24.5 ± 0.5 °C. The water used as a subphase was redistilled from an all-quartz system. The subphases were tested for surface active impurities by exposing a large area of subphase surface for about 1 hr and then compressing any impurity film to a small area. The lowering of the subphase surface tension due to impurities was negligible. The spreading solvents, benzene and chloroform, were twice distilled and tested for active impurities by spreading on clean subphases.

The polymer solutions were spread from micropipets. Ten minutes was usually allowed for solvent evaporation before compressing the polymer films. This time was considered ample as the surface pressure became constant within 5 min after spreading. In a few instances the films were allowed to stand for 30 min to check for pressure changes. No changes were observed. Concentrated solutions of poly(pentamethylene succinate), however, did not reach constant film pressures in the time interval of 10 min, but gave steadily increasing pressures for longer periods of time. Dilute solutions of this polymer, however, spread in the usual manner.

The properties of the completely spread films were not sensitive to the film compression rate. The average time of an experimental run was 1 hr.

One experiment was run at a liquid-liquid interface with a verticle pull film balance, frequently known as a Wilhelmy type balance [5]. A plate is suspended vertically from one arm of an analytical balance and is partially immersed in a liquid. The force on the balance arm is determined first with the pure liquid and then with the liquid on which a film has been spread. In this manner differences in the surface tension and therefore the film pressure are determined. The nature of this type of film balance also permits it to be used with a film at a liquid-liquid interface. The plate, a cleaned glass microscope slide, was held with a polytetrafluoroethylene block that hung from the arm of an analytical balance by means of a platinum wire. The water phase was prepared as described above and the other phase, cyclohexane, was of spectral grade. Both liquids were added after the plate was suspended in place, with approximately one-half of the plate suspended in each liquid. The plate was moved slightly from the aqueous phase into the organic phase to determine the reference point. The polymer in benzene solution was then added with a micropipet in successive increments at the interface. The pressure was determined after each increment.

## 3. Results

**Figure 1:**
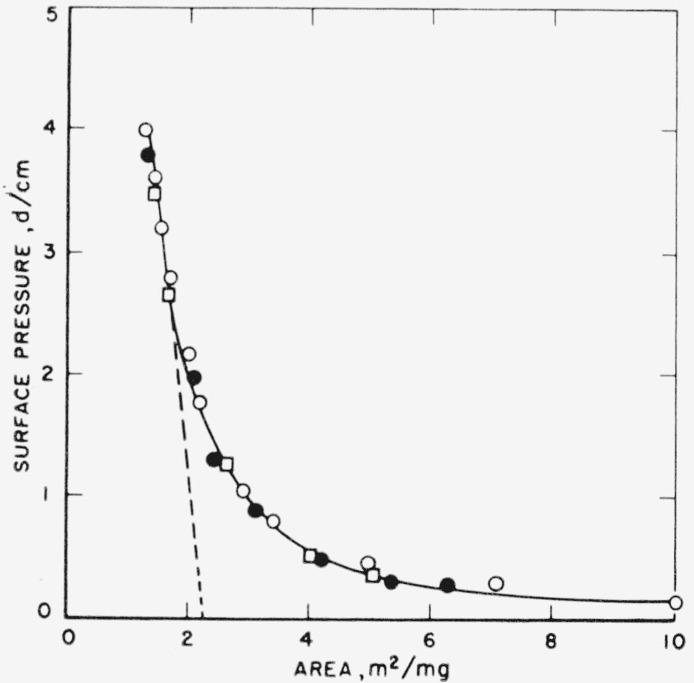
Surface pressure-area isotherm of poly (ethylene succinate) on aqueous subphases at 24.5 ± 0.5 °C. Subphase – Distilled water ● 5.51×10^−3^ mg □ 11.02×10^−3^ mg Subphase – 0.01 N HCl ○ 5.51×10^−3^ mg

**Figure 2:**
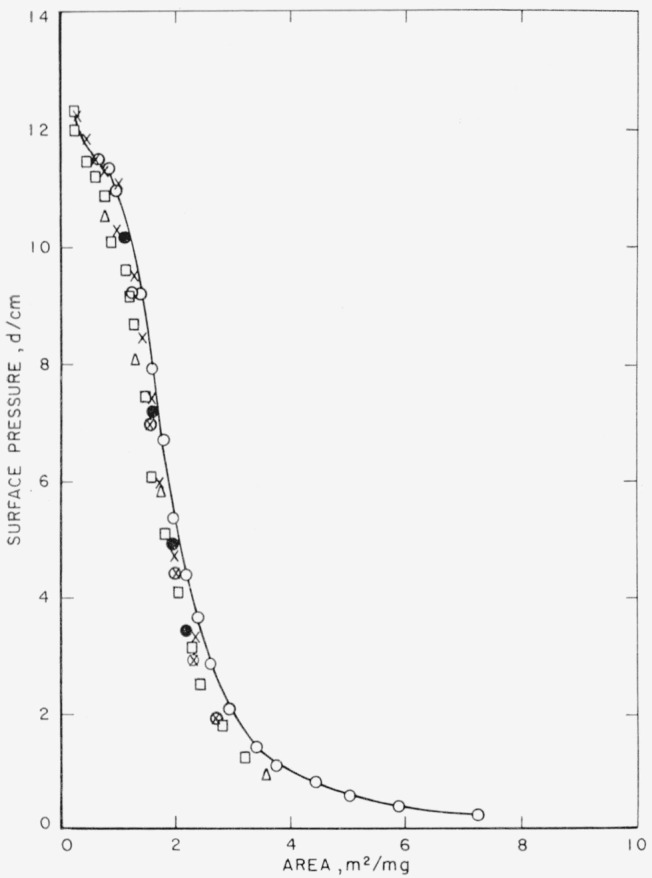
Surface pressure-area isotherms of poly(pentamethylene succinate) on distilled water at 24.5 ± 0.5 °C. Spread from chloroform ○ 9.56×10^−3^ mg, Run 1 ● Points obtained on expansion of film of Run 1 □, Δ 19.12×10^−3^ mg Spread from benzene ×, ⊗ 24.46×10^−3^ mg

**Figure 3:**
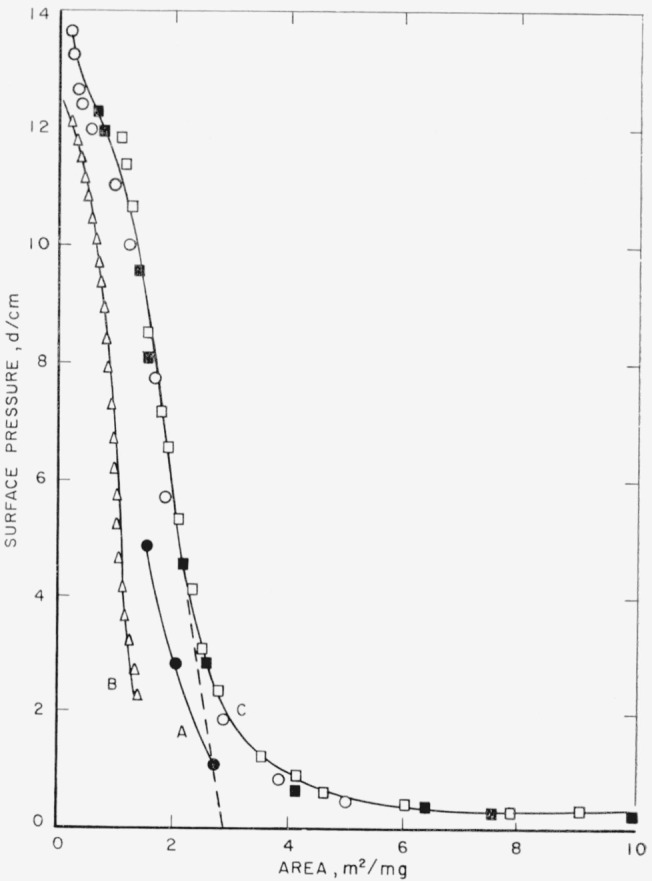
Surface pressure-area isotherm of poly(pentamethylene succinate) spread from benzene at 24.5 ± 0.5 °C. Curves A and B—Distilled water subphase ● 24.96×10^−3^ mg Δ 48.92×10^−3^ mg Curve C—0.01 N HCl subphase □, ■ 5.59×10^−3^ mg ○ 11.18×10^−3^ mg

**Figure 4:**
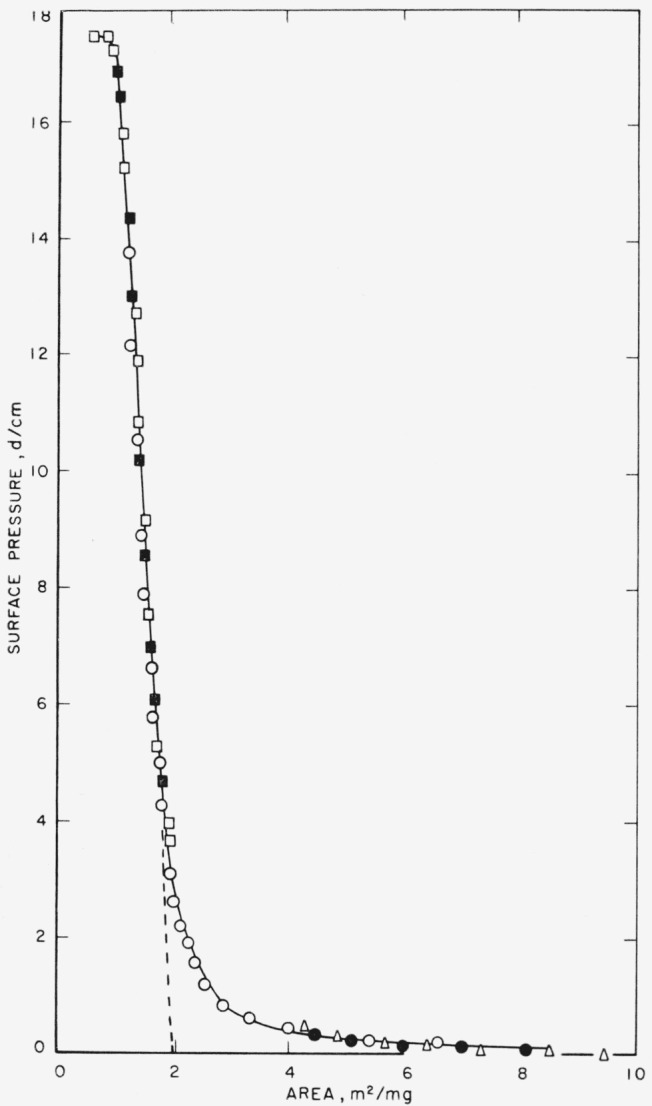
Surface pressure-area isotherm of poly (neopentyl succinate) on distilled water subphases at 24.5 ± 0.5 °C. Spread from chloroform ○ 10.40×10^−3^ mg Spread from benzene ●, Δ 5.0×10^−3^ mg □, ■ 30.76×10^−3^ mg

**Figure 5:**
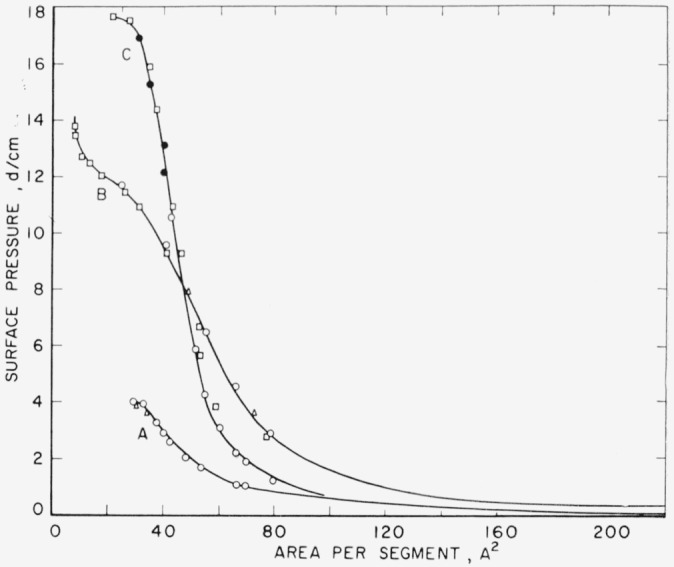
Surface pressure-area isotherms of linear saturated polyesters on aqueous subphases at 24.5 ± 0.5 °C. Curve A Poly(ethylene succinate) Curve B Poly(pentamethylene succinate) Curve C Poly(neopentyl succinate)

Pressure-area isotherms were determined with the horizontal balance. They are given in figures 1 to 5.

Figure 1 gives the monolayer properties of poly (ethylene succinate) spread from chloroform on distilled water and 0.01 *N* hydrochloric acid subphases. The isotherm shows the surface pressure, expressed as dynes/cm, as a function of the specific area of the polymer. Increasing the volume of solution spread at constant subphase area by a factor of 2 did not alter the film properties. The isotherm also shows the film properties to be independent of subphase *p*H for values between 2 for HCl solutions and 6.5 for water. Also of interest is the highly expanded state and compressibility of these films. No linearity in the isotherm was observed until pressures slightly below collapse were obtained. An extrapolation from a region of least curvature gives a limiting specific area of approximately 2.3 m^2^ per milligram. The absence of any change in specific area with volume of solution suggests that these films were completely spread and probably existed as monolayers.

The spreading characteristics and specific areas of poly(pentamethylene succinate) films are given in figures 2 and 3. The isotherm showing the film properties of poly (pentamethylene succinate) spread from chloroform on distilled water is given in figure 2. The curve is drawn through the points obtained with 9.56×10^−3^ mg of polymer and 50 λ (0.050 ml) of solution. Films spread from dilute benzene solutions on 0.01 N hydrochloric acid subphases show curves that are identical with the curve drawn on this figure. The isotherm for this polymer spread on the hydrochloric acid subphase is given by curve C in figure 3.

Poly (pentamethylene succinate) was particularly sensitive to the quantity of material spread. Solutions of increasing concentration were spread to produce films, some of which when completely spread would approach or exceed the available surface area on the balance. The area required for complete spreading was determined from the specific area. The available surface area on the balance at the time of spreading was approximately 700 cm^2^. Within experimental error the same curve was obtained up to quantities of 11.18×10^−3^ mg, corresponding to an area of approximately 325 cm^2^, as shown by curve C of figure 3. Quantities of 19.12×10^−3^ mg, corresponding to an area of approximately 555 cm^2^, however, resulted in a slight shift in the isotherm. This is shown in figure 2 where the drawn curve is through the points obtained from 9.56×10^−3^ mg. The points determined from the 19.12×10^−3^ mg sample lie to the left of this drawn curve. Although not completely spread, duplicate runs with this quantity resulted in a reproducible isotherm. Incomplete, but apparently reproducible spreading was also observed when 24.46×10^−3^ mg were used. This quantity of material would produce a film with an area of approximately 700 cm^2^, the limiting area of the tray. Although the spreading was not complete, there was again only a small shift from the true isotherm for complete spreading, which yields the greatest area for a given pressure.

Larger quantities of polymer resulted in progressively larger shifts in the isotherm, as shown by curves A and B in figure 3. In the case of isotherm B the available surface area on the balance was approximately one-half of that required by the film. In all cases any incomplete spreading resulted in apparent low specific areas.

When monolayer investigations are conducted with polymers, adequate attention must be given to assure complete spreading of the polymer films. The use of a single concentration may give reproducible films with reasonable area values, even though the films may still be incompletely spread.

The isotherm drawn in figure 2 and isotherm C in figure 3 were selected as those representing the film characteristics of fully spread poly(pentamethylene succinate) films on distilled water and acidic subphases. The usual inflection was exhibited, but on further compression collapse did not occur. Instead, the film assumed a more compressible state and eventually passed into a state resistant to compression. The extrapolated specific area for the completely spread polymer film was 2.9m^2^ per milligram. The reversibility of the area occupied by the film of this polymer is shown in figure 2 by the closed circles. They were obtained by compressing the film through the inflection point (open circles), followed by expansion (closed circles).

The isotherm of poly (neopentyl succinate), an isomer of poly(pentamethylene succinate), is given in figure 4. This polymer differs from its isomer in the arrangement of the atoms in the glycol group. The lower portion of this isotherm was obtained using a sensitive torsion wire and the dilute benzene-polymer solutions. These are shown by the closed circles and open triangles. The highest recorded surface pressure for this range was about 0.4 dynes per centimeter. When the solution concentration was increased threefold (to 0.3 mg/ml) and the volume of solution spread twofold (to 100 λ), the isotherms produced were reproducible and gave the curve drawn through the open and closed squares. The lower portion of this isotherm is higher than that obtained for the low pressure studies.

To ascertain the intermediate portion of the isotherm, as well as to determine the effect of spreading-solvent on the film state, films were spread with 100 λ of chloroform solutions of 0.1-mg/ml polymer concentration. This resulted in a quantity of polymer on the film balance intermediate between the two other quantities studied. The experimental points obtained are shown by the open circles, and these points overlapped both regions previously studied. The experimental agreement was very good. The isotherm derived from these studies indicates that the spreading properties of poly (neopentyl succinate) films were independent of these two spreading solvents. The spreading was not very sensitive to the quantity of polymer deposited as the 30.76×10^−3^ mg corresponded to an area of approximately 615 cm^2^, based on the extrapolated specific area, approaching the available film balance area of 700 cm^2^. Therefore, the isotherm shown is that for the completely spread films of this polymer on distilled water subphases.

Approximate surface film thicknesses were calculated from the extrapolated specific areas and the respective bulk densities of the polymers. These film thicknesses are given in table 2. The thickness values ranging from 3 to 4 A indicate that the completely spread films are monomolecular. It appears that molecules of these linear succinate polymers lie flat on the subphase surface in the same manner as the molecules of the self ester of *ω*-hydroxydecanoic acid reported by Harkins and coworkers [1]. The bulk densities of the polymers are given in table 1 and the extrapolated areas of the isotherms to zero surface pressure in table 2. Also listed in table 2 are the calculated and observed unit areas, collapse pressures, energies to compress the films to collapse, and film compressibilities.

The compressibilities of the films were calculated using the following equation:

$$K=\frac{A_{0}-A_{1}}{A_{0}\pi_{1}}$$

where *K* is the compressibility; *A*_0_ the extrapolated specific area of closely packed molecules at zero surface pressure; and *A*_1_ the specific area at surface pressure *π*_1_.

The stability of the polymer films depends on both the lateral cohesion between the chains and the energy required to pull hydrophilic groups from the aqueous subphase. The energy required to compress the film to the point of collapse or to the inflection point in the case of poly (pentamethylene succinate) is obtained by integrating the area under the isotherm up to the point of collapse or inflection. The energy required to compress the film is given by:

$$\Delta E=\int_{A_{0}}^{A_{i}}\pi_{s}dA$$

where δ*E* is the energy necessary to compress the film; *A*_0_, a very large segment area at which the surface pressure is very small; and *A_i_*, the segment area at the point at which film instability occurs. The energies obtained from the isotherms by graphical integration are given in table 2.

## 4. Discussion

Monolayers of linear polymers having carboxyl groups have been studied by Harkins, Carmen, and Ries [1], Crisp [6], and others. The results of these investigations show the surface pressure-area isotherms of the films to be essentially independent of the number of repeating units per molecule and, therefore, the molecular weight of the polymer. This independence of molecular weight permits a more meaningful analysis of monolayer properties in terms of the fundamental repeating structural unit.

The polymer films, therefore, were assumed to be completely spread and the isotherms independent of molecular weight. The experimental data are replotted in figure 5 to express the surface pressure as a function of the area in A^2^ occupied by a single polyester unit. This figure shows the monolayer properties of the three polyesters on these aqueous subphases. Some experimental points of different runs are included to indicate reproducibility.

Assumed interfacial conformations of the three polymers can be represented by means of molecular models. The plane of the zigzag of the molecules is parallel to the surface of the substrate. The neopentyl group is a rigid, bulky mass that probably lies more above the other portions of the molecule than do the ethylene or pentamethylene groups in the other polymers. In the latter the methylene groups of the glycol can lie in the same plane and in contact with the surface.

The molecules of poly (ethylene succinate) may be considered as long chain normal paraffins into which ester linkages have been periodically placed. These polar ester groups are most likely primarily responsible for the binding and spreading characteristics of this polymer. Presumably the carbonyl groups of the ester linkages are oriented towards the aqueous phase.

The monolayer of poly (ethylene succinate), as shown by isotherm A in figure 5, is highly expanded and exhibits no linearity in the region approaching collapse pressure. Extrapolating from an ambiguous linear portion of the isotherm, a limiting unit area of 60 to 70 A^2^ is obtained. This observed area is larger than that expected from the fiber diagram of this polymer as determined by Fuller and Erickson [7] using X-ray methods. The observed area, however, agrees quite well with that reported by Moss [2] for the same polymer of 3200 number average molecular weight. Molecular models show that the unit length can vary little when completely spread and oriented. The large areas observed must then be caused by a loose packing of the film molecule. Another striking feature is the high compressibility of this monolayer, 0.11 cm/dyne (table 2). The low collapse pressure observed, 4 dynes per centimeter, is reproducible and much higher than the value of 2.5 dynes per centimeter reported by Moss [2] for monofilms of this polymer.

The marked effect on the monolayer properties produced by the addition of three methylene groups is seen by comparing isotherms A and B in figure 5. As in the case of the poly (ethylene succinate) monolayer, the monolayer of poly(pentamethylene succinate) is expanded and compressible, having a compressibility of 0.056 cm/dyne (table 2). The monolayer of poly(pentamethylene succinate) does not collapse when compressed to small areas. Instead, at areas where collapse is expected to occur the film becomes quite compressible, presumably passing into a multilayer or crumpled structure. Further compression of the film produces another inflection in the isotherm. At this specific area the film is probably passing into a multimolecular structure resistant to compression, as shown by an upswing of isotherm B, figure 5. Similar monolayer and film characteristics have been observed for other polymers such as some poly (alkyl methacrylates) as described by Crisp [8] and some polyorganosiloxanes as reported by Fox, Taylor and Zisman [9]. The extrapolated repeating unit area of poly(pentamethylene succinate), 90 A^2^, is about 20 percent larger than that expected for the unit in the crystalline state.

The monolayer properties of poly (neopentyl succinate) on distilled water are given by isotherm C in figure 5. The curve is less expanded over the entire surface pressure range than those of the other polymers. It is also less compressible, having a compressibility of 0.028 cm/dyne (table 2), and collapses at 17.7 dynes per centimeter. This collapse occurs at a segment area of about 25 A^2^. It is interesting to note that the extrapolated repeating unit area, 63 A^2^, is very close to that expected for a unit of the polymer in an oriented crystalline state.

**Figure 6:**
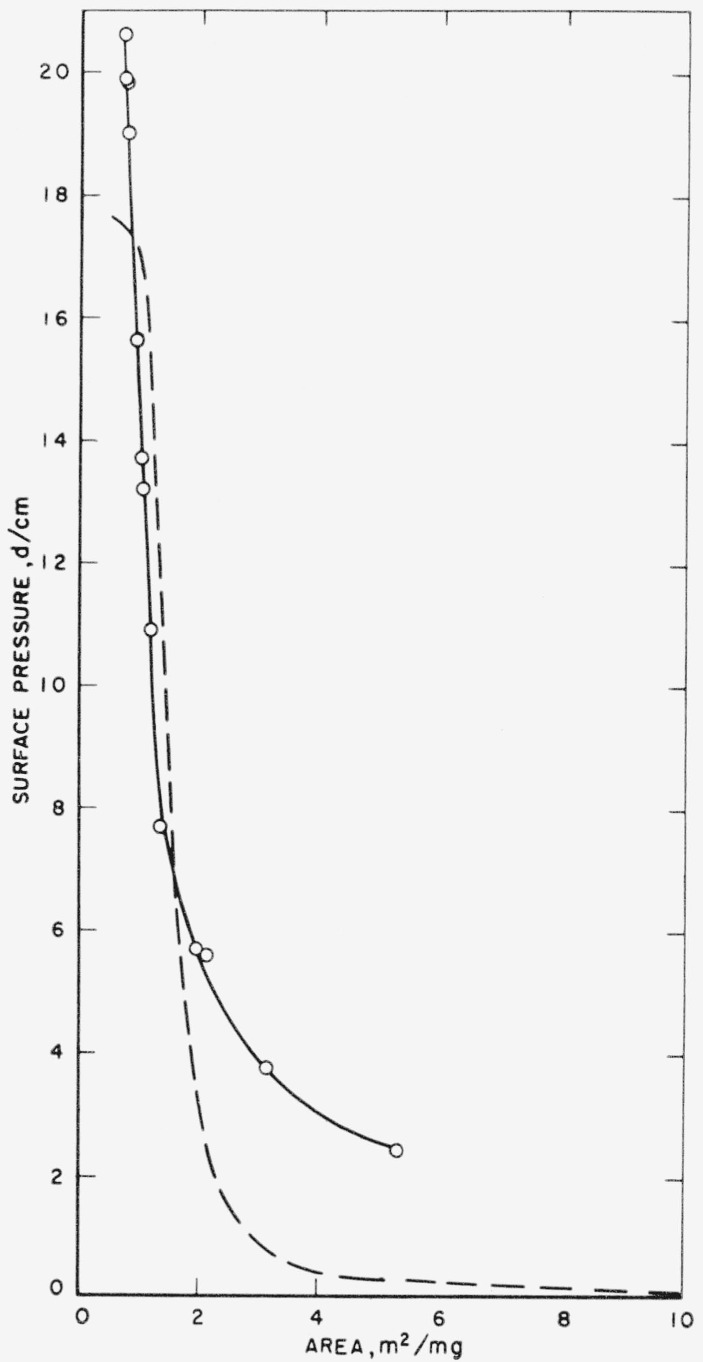
Surface pressure-area isotherms of poly (neopentyl succinate) at air-water and liquid-liquid interfaces. Polymer spread from benzene ○ cyclohexane-water interface - - - air-water interface

The low expansion and compressibility of the poly (neopentyl succinate) monolayer is attributable to the inflexibility of the unit produced by the presence of the rotationally highly hindered neopentyl group. The surface pressure-area isotherm of this polymer at the cyclohexane-water interface, determined with a Wilhelmy balance, is given in figure 6. It shows that the high surface pressure region of the isotherm is essentially the same as that for the air-water interface. At low pressures, however, there is evidence of a more expanded film at the oil-water interface as compared to that at the air-water interface. At high surface pressures the absence of film expansion at the oil-water interface suggests that the expansion of the monolayer at low pressures is not caused by changes in intermolecular forces but by chain rigidity.

## 5. Summary

The monolayer properties of the succinate polyesters were found to be closely related to structural features of the glycols used. This was shown in a marked manner by the differences in the interfacial properties of poly (pentamethylene succinate) and poly (neopentyl succinate). The repeating units of these two polymers are isomeric. The extrapolated unit area was larger for the poly (ethylene succinate) and poly(pentamethylene succinate) than that calculated for a unit of the polymer in the crystalline state. For the semirigid poly (neopentyl succinate) unit the observed and calculated areas were about the same.

## Figures and Tables

**Table 1:** Properties of the polyesters

Polymer	Softening point	Molecular weight ${\bar{M}}_{n}$^a^	Bulk density^b^ at 27 °C
			
	°*C*		*g/cm*^3^
Poly (ethylene succinate)	96	4100	1.4
Poly (pentamethylene succinate)	38	4500	1.2
Poly (neopentyl succinate)	76	4400	…………………………

a ${\bar{M}}_{n}$ = number average molecular weight determined from supplied end group analysis.

b Determined by method of hydrostatic weighings.

**Table 2:** Monolayer properties of some succinate polyesters on aqueous subphases

Polymer	Extrapolated specific area	Area per seg.		Collapse pressure	Energy to compress film to collapse	Compressibility	Thickness at *π* = 0
		Calc.	Obs.				
							
	*m^2^/mg*	*A*^2^	*A*^2^	*dynes/cm*	*cal. mole*^−1^ *segment*^−1^	*cm/dyne*	*A*
Poly (ethylene/succinate)	2.3	55	60 to 70	4.0	349	0.11	3.1
Poly (pentamethylene succinate)	2.9	74	90	Plateau (11.7)	568^a^	.056	2.9
Poly (neopentyl succinate)	2.0	60	63	17.7	734	.028	3.9

a Energy to compress film to midpoint of first inflection in isotherm.

## Footnotes

4 Figures in brackets indicate the literature references at the end of this paper.
